# ESMO 2020: highlights in breast cancer

**DOI:** 10.1007/s12254-021-00713-5

**Published:** 2021-04-30

**Authors:** Rupert Bartsch

**Affiliations:** Department of Medicine 1, Division of Oncology, Medical University of Vienna, Waehringer Guertel 18–20, 1090 Vienna, Austria

**Keywords:** ESMO Congress 2020, Breast cancer, Highlights, ASCENT, Atezolizumab, MonarchE, PALLAS, Review

## Abstract

Despite the ongoing severe acute respiratory syndrome coronavirus 2 (SARS-CoV-2) pandemic, results of several pertinent studies in the field of breast cancer (BC) were presented in a virtual format at the 2020 European Society of Medical Oncology (ESMO) Congress. Early results of the MonarchE trial investigating the addition of the cyclin-dependent kinase (CDK) 4/6 inhibitor abemaciclib to standard adjuvant endocrine therapy indicated a lower recurrence rate in the combination group in a high-risk population of patients with early stage hormone receptor (HR)-positive/HER2-negative BC. In contrast, the PALLAS study evaluating adjuvant palbociclib could not confirm these results. Subtle differences in the respective trial populations, a higher discontinuation rate in PALLAS, or substance-specific differences may be responsible. In HER2-positive early stage BC, long-term results of the ADAPT-TP trial support the notion that chemotherapy-free treatment may be possible in a subset of patients with favourable response to HER2-directed therapy without compromising long-term outcome. The phase III IMpassion031 trial evaluated the addition of atezolizumab to neoadjuvant anthracycline/taxane-containing chemotherapy in triple-negative BC (TNBC). A significant improvement in terms of pathologic complete remission rate was observed but data concerning long-term outcome must be awaited. Final overall survival (OS) analysis of IMpassion130 confirmed the clinically relevant OS improvement observed with the addition of atezolizumab to first-line nab-paclitaxel in metastatic PD-L1 positive TNBC. In contrast, no benefit was observed with the addition of atezolizumab to solvent-based paclitaxel in a similar population. This contradiction is commonly explained by the need for corticosteroid co-medication with conventional paclitaxel, but the exact reason remains poorly understood. Antibody–drug conjugates (ADCs) have been successfully established in HER2-positive breast cancer; in TNBC, the phase III ASCENT trial compared the ADC sacituzumab govitecan with chemotherapy by physician’s choice in pretreated metastatic patients. A significant improvement in terms of progression-free survival and OS was observed rendering this drug a potential novel standard in this patient population.

## Introduction

After the virtual ASCO Annual Meeting, the 2020 Congress of the European Society of Medical Oncology (ESMO) was also held in a virtual format due to the ongoing severe acute respiratory syndrome coronavirus 2 (SARS-CoV-2) pandemic. Despite this drawback, long-awaited data from several important trials in the field of breast cancer (BC) were presented. This article is intended as a short review of these results.

## MonarchE and PALLAS

Inhibitors of the cyclin-dependent kinases 4 and 6 (CDK4/6) when added to endocrine therapy resulted in a major improvement of outcome in patients with hormone receptor (HR)-positive/HER2-negative metastatic BC. Based upon these results, trials evaluating the potential role of these drugs in the adjuvant setting were initiated. At these year’s ESMO Congress, results of two studies—MonarchE and PALLAS—were presented [1–3].

MonarchE accrued a total of 5637 patients with early stage breast cancer; patients were randomized 1:1 to standard-of-care endocrine therapy (ET) alone or the combination of ET with abemaciclib administered for a total duration of 2 years. Patients had to have high recurrence risk, defined as ≥ 4 involved lymph nodes; in case of 1–3 lymph involved nodes, a tumour size of at least 5 cm, and/or histologic grade 3 disease, and/or a proliferation rate of ≥ 20% were required. At a median follow-up of 15.5 months, estimated 2‑years invasive disease-free survival (which was defined as the primary study endpoint) was improved from 88.7% in the control group to 92.2% (hazard ratio [HR] 0.75; 95% confidence interval [CI] 0.60–0.93; *p* = 0.01) with the addition of abemaciclib; no differential effects were observed in the relevant subgroups. As expected, the most common side-effect in the abemaciclib group was diarrhoea (grade 3 diarrhoea 7.6%; any grade diarrhoea 82.2%); apparently, this toxicity was manageable as the highest rates of grade 2/3 diarrhoea was observed in the first treatment months and decreased thereafter. Overall, 16.6% of participants discontinued abemaciclib due to toxicity. At data cut-off, 72.8% of patients were still within the 2‑year treatment phase.

While similar at first glance, the PALLAS trial brought contradicting results. Here, 5760 patients with early stage HR-positive/HER2-negative breast cancer were randomized to standard ET plus additional adjuvant therapy with palbociclib or control. At a median follow-up of 23.7 months, invasive disease-free survival was comparable in both arms (HR 0.93; 9%% CI 0.76–1.15). Of note, patients at lower recurrence risk were included into PALLAS (18% clinical stage IIA; 13% N0), and early discontinuation rates were higher compared with MonarchE (early discontinuation rate overall 42.2%; early discontinuation rate due toxicity 27.1%). The latter observation may be due to an earlier start of PALLAS when investigators had less clinical experience with CDK4/6 inhibitors and the longer follow-up.

Therefore, contradicting outcomes in the two studies may be explained by the mentioned subtle differences in patient characteristics, the lower discontinuation rate in MonarchE, or drug-specific differences. Updated results of the MonarchE trial are awaited at the (virtual) 2020 San Antonio Breast Cancer Symposium; if a continued benefit—or even a larger separation of the curves—is observed, abemaciclib is expected to become an additional adjuvant treatment option in luminal breast cancer at high recurrence risk.

## ADAPT-TP

Neoadjuvant treatment is regarded as standard-of-care for the majority of patients with early stage HER2-positive BC; with high pathologic complete remission (pCR) rates observed, several studies have concentrated on strategies of chemotherapy de-escalation. The ADAPT-TP trial was a phase II study comparing 12 weeks of neoadjuvant T‑DM1 with T‑DM1 plus endocrine therapy and trastuzumab plus endocrine therapy in 375 luminal B/HER2-positive early stage BC patients. The primary outcomes were already published, with 41.0% of patients in the T‑DM1 group, 41.5% of patients receiving T‑DM1 and ET, and 15.1% on trastuzumab and ET achieving pCR [4]. At the 2020 ESMO conference, data regarding long-term outcome were presented [5]. As expected, pCR translated into improved disease-free survival (DFS) at 5 years on a patient level (92.7% vs. 82.7%; *p* = 0.014) while due to the size and design of the study, no significant DFS difference was observed between the three treatment arms. Out of 117 patients with pCR, 76 received postoperative chemotherapy while 41 did not; at 5 years, no DFS difference was seen between these groups. Although further confirmation is required, these data suggest that in patients with high sensitivity to HER2-directed treatment withholding chemotherapy without compromising long-term outcome may be possible.

## IMpassion031

In stage II and III triple-negative BC (TNBC), neoadjuvant administration of chemotherapy is the preferred treatment option and trials have focused on novel strategies to improve pCR rates. In the phase III KENYOTE-522 trial, addition of the immune checkpoint inhibitor pembrolizumab to quadruple chemotherapy (paclitaxel/carboplatin followed by epirubicin/cyclophosphamide) yielded a significant pCR increase while data regarding event-free survival (EFS) are still awaited [6]. In contrast, no significant pCR improvement was seen with the addition of the PD-L1 inhibitor atezolizumab to a nonstandard regimen consisting of eight cycles of neoadjuvant nano-albumin-bound (nab) paclitaxel/carboplatin [7]. In the phase III IMpassion 031 study, atezolizumab or placebo was added to neoadjuvant nab-paclitaxel followed by doxorubicin/cyclophosphamide in 333 patients [8, 9]. Addition of immunotherapy resulted in a significant improvement of pCR rate from 41% to 58% (∆pCR 17%; 95% CI 6–27; *p* = 0.0044), which was defined as the primary study endpoint. The benefit was observed independently of the PD-L1 status and pCR rates were higher in PD-L1-positive patients in both treatment arms. These data are well in line with results from KEYNOTE-522 and suggest that in contrast to metastatic TNBC, PD-L1 positivity is not required as predictive biomarker in early stage disease but rather describes a phenomenon of immunogenicity resulting in a general improvement of treatment activity.

Therefore, atezolizumab and pembrolizumab were both shown to increase pCR rates when added to sequential taxane/anthracycline-based chemotherapy meanwhile. If a benefit regarding long-term outcome is confirmed, it is expected that immunotherapy will become a standard component of neoadjuvant therapy for high-risk TNBC.

## IMpassion130 and IMpassion 131

IMpassion 130 was a placebo-controlled prospective randomized phase III trial evaluating the role of atezolizumab when added to nab-paclitaxel as first-line treatment for metastatic (m) TNBC. In the primary analysis, combination treatment yielded a clinically relevant albeit formally not significant prolongation of overall survival (OS) from 15.5 to 25.0 months (HR 0.62; 95% CI 0.45–0.86) in the PD-L1-positive subset and these outcomes have led to the approval of atezolizumab in combination with nab-paclitaxel as first-line treatment of PD-L1-positive mTNBC [10]. Data from the final OS analysis at a median follow-up of 18.8 months were presented at the 2020 ESMO Congress confirming previous results [11]: In the ITT population, a trend towards improved OS was observed, with a prolongation of OS from 18.7 to 21.0 months (HR 0.87; 95% CI 0.75–1.02; *p* = 0.077); this difference was again driven by the PD-L1-positive subset (OS 17.9 vs. 25.4 months; HR 0.67; 95% CI 0.53–0.86) while no benefit was observed in PD-L1 negative tumours.

IMpassion131, in contrast, used conventional (solvent-based) paclitaxel as chemotherapy backbone [12]. In total, 651 patients with locally advanced, inoperable, or mTNBC without prior treatment for advanced disease were randomized 2:1 to atezolizumab or placebo plus paclitaxel. Investigator-assessed progression-free survival (PFS) was defined as the primary study endpoint, and no difference was observed between the treatment groups in the PD-L1-positive subset (PFS PD-L1 positive 5.7 months atezolizumab/paclitaxel vs. 6.0 months placebo/paclitaxel; HR 0.82; 95% CI 0.6–1.12) as well as in the ITT population (5.6 vs. 5.7 months; HR 0.86; 95% CI 0.70–1.05). No differences in terms of OS were observed as well.

The exact reasons for these contradicting results are currently not well understood but usually the corticosteroid comedication required for the administration of conventional paclitaxel is held responsible. For the time being, nab-paclitaxel plus atezolizumab remains the standard-of-care in this patient population.

## ASCENT

Sacituzumab govetican is an antibody–drug conjugate (ADC) consisting of a monoclonal antibody targeting the tumour-associated calcium signal transducer 2 (trophoblast cell-surface antigen 2; Trop-2) protein, a surface protein commonly overexpressed in TNBC, and SN-38, the active metabolite of irinotecan. Based upon promising phase II data [13], the drug has meanwhile received accelerated approval by the US Food and Drug Administration for the use in pretreated mTNBC patients [14].

In the phase III ASCENT trial, sacituzumab govitecan was compared to chemotherapy by physician’s choice (capecitabine, gemcitabine, eribulin, vinorelbine) in 529 patients with mTNBC who had received at least two prior treatment lines for metastatic disease [15]. Of note, more than 25% of participants had already received immunotherapy with checkpoint inhibitors. A statistically significant and clinically relevant benefit in terms of PFS was observed in the sacituzumab govitecan group (PFS 5.6 vs. 1.7 months; HR 0.41; 95% CI 0.32–0.52; *p* < 0.0001) which translated into a doubling of overall survival (OS) (12.1 vs. 6.7 months; HR 0.48; 95% CI 0.38–0.59; *p* < 0.0001). Regarding toxicity, higher rates of grade 3/4 neutropenia and febrile neutropenia were observed in the experimental arm (63% vs. 40% and 6% vs. 2%, respectively). In addition, 10% of patients experienced grade III diarrhoea on the ADC with numerically higher numbers of grade 3 nausea and vomiting also observed. These side-effects were apparently manageable and therefore, sacituzumab govitecan is a highly promising drug that will see broad use in mTNBC once available in Europe.

### Take home message

- Early results of the MonarchE trial suggest a reduction of recurrence risk with the addition of abemaciclib to endocrine therapy in patients with high-risk HR-positive/HER2-negative early stage BC but longer follow-up is required. In the neoadjuvant setting, long-term results of ADAPT-TP supports the hypothesis that foregoing standard chemotherapy may be feasible in patients with excellent response to neoadjuvant HER2-directed treatment. In line with previous results, a clinically relevant improvement of pCR rates was observed with the addition of immunotherapy to standard neoadjuvant chemotherapy in the IMpassion031 trial while data regarding long-term outcome are still immature.
- In metastatic TNBC, the final overall survival analysis of IMpassioon130 confirmed the survival benefit obtained with the addition of atezolizumab to nab-paclitaxel as first-line treatment of PD-L1-positive disease; in contrast, no benefit was obtained with the addition of atezolizumab to solvent-based paclitaxel. Finally, the phase III ASCENT trial established the superiority of sacituzumab govitecan over conventional chemotherapy in pretreated mTNBC patients.

## References

[1] Johnston SRD, Harbeck N, Hegg R (2020). Abemaciclib in high risk early breast cancer. Ann Oncol.

[2] Johnston SRD, Harbeck N, Hegg R (2020). Abemaciclib combined with endocrine therapy for the adjuvant treatment of HR+, HER2−, node-positive, high-risk, early breast cancer (monarchE). J Clin Oncol.

[3] Mayer EL, Gnant MI, DeMichele A (2020). PALLAS: a randomized phase III trial of adjuvant palbociclib with endocrine therapy versus endocrine therapy alone for HR+/HER2− early breast cancer. Ann Oncol.

[4] Harbeck N, Gluz O, Christgen M (2017). Adjusted personalized therapy trial optimizing risk assessment and therapy response prediction in early BC HER2− and hormone receptor-positive phase II randomized trial-efficacy, safety, and predictive markers for 12 weeks of Neoadjuvant Trastuzumab Emtansine with or without endocrine therapy (ET) versus Trastuzumab plus ET. J Clin Oncol.

[5] Harbeck N, Nitz U, Christgen M (2020). De-escalated neoadjuvant T-DM1 with or without endocrine therapy (ET) vs trastuzumab+ET in early HR+/HER2+ breast cancer (BC): ADAPT-TP survival results. Ann Oncol.

[6] Schmid P, Cortes J, Pusztai L (2020). Pembrolizumab for early triple-negative breast cancer. N Engl J Med.

[7] Gianni L, Huang C, Egle D (2019). Pathologic complete response to neoadjuvant treatment with or without atezolizumab in triple-negative, early high-risk and locally advanced breast cancer. NeoTRIPaPDL1 Michelangelo randomized study.

[8] Harbeck N, Zhang H, Barrios CH (2020). IMpassion031: results from a phase III study of neoadjuvant (neoadj) atezolizumab + chemotherapy in early triple-negative breast cancer (TNBC). Ann Oncol.

[9] Mittendorf EA, Zhang H, Barrios CH (2020). Neoadjuvant atezolizumab in combination with sequential nab-paclitaxel and anthracycline-based chemotherapy versus placebo and chemotherapy in patients with early-stage triple-negative breast cancer (IMpassion031): a randomised, double-blind, phase 3 trial. Lancet.

[10] Schmid P, Adams S, Rugo HS (2018). Atezolizumab and nab-paclitaxel in advanced triple-negative breast cancer. N Engl J Med.

[11] Emens LA, Adams S, Barrios CH (2020). IMpassion130: Final OS analysis from the pivotal phase III study of atezolizumab + nab-paclitaxel vs placebo + nab-paclitaxel in previously untreated locally advanced or metastatic triple-negative breast cancer. Ann Oncol.

[12] Miles DW, Gligorov J, Andre F (2020). Primary results from IMpassion131, a double-blind placebo-controlled randomised phase III trial of first-line paclitaxel (PAC) ± atezolizumab (atezo) for unresectable locally advanced/metastatic triple-negative breast cancer (mTNBC). Ann Oncol.

[13] Bardia A, Mayer IA, Vahdat LT (2019). Sacituzumab govitecan-hziy in refractory metastatic triple-negative breast cancer. N Engl J Med.

[14] https://www.fda.gov/drugs/drug-approvals-and-databases/fda-grants-accelerated-approval-sacituzumab-govitecan-hziy-metastatic-triple-negative-breast-cancer. Accessed 30 Nov 2020.

[15] Bardia A, Tolaney SM, Loirat D (2020). ASCENT: A randomized phase III study of sacituzumab govitecan (SG) vs treatment of physician’s choice (TPC) in patients (pts) with previously treated metastatic triple-negative breast cancer (mTNBC). Ann Oncol.

